# Book Reviews

**Published:** 1810-05

**Authors:** 


### CRITICAL ANALYSIS
### OF THE
### RECENT PUBLICATIONS
### IN THE
### DIFFERENT BRANCHES OF PHYSIC, SURGERY, AND MEDICAL PHILOSOPHY.

*A Scientific and Popular View of the Fever of Walcheren and its Consequences, as they appeared in the British Troops returned from the late Expedition; with an Account of the Morbid Anatomy of the Body, and the Efficacy of Drastic Purges and Mercury in the Treatment of this Disease.* By J. B. DAVIS, M. D. one of the Physicians appointed by the Medical Board to attend the Sick Troops returned to England. 8vo. 224 pages. London.

A MORE important subject than the fever of Walcheren cannot well be fixed upon by the physician for investigation, whether he considers it with respect to its source and origin in soil and climate, in order successfully to resort to prophylactic means against its invasion, or examines the nature, tendencies, and diversified phenomena of the fever itself, both with a view to reduce it to a mild and manageable type, as well as to avert the complicated and fatal consequences of one of the direst diseases that ever appeared in any temperate region. Dr. Davis has imposed upon himself a task of difficulty, though of great public moment, in undertaking to lay before the world an account of a disease, that has, from its unexampled violence and fatality, called forth the attention of men distinguished in medical science. Nevertheless, he has accomplished his object with that success and credit to himself, which cannot fail of making his work a valuable and acceptable present to others. It is necessary, for the information of every practitioner in our own age, age, and of ages to come, to be in possession of a record of the Walcheren fever and its consequences, both with a view to trace its analogy with the fevers of the marshes of Lincoln, Kent, and Essex, and to judge of the efficacy of certain remedies for their removal. Sir John Pringle and Cleghorn have described the symptoms and appearances of remitting and intermitting fevers with accuracy, but they are both defective in minutiæ, and, above all, in that very interesting and useful part, the dissections, which our author presents us with in a particular and general manner. In this interesting work, we have pleasure in saying that we discover skill, perspicuity of expression, and a spirit of investigation, which pursues its aim with energy. In it we moreover find many luminous views of several diseases arising out of the primary fever, and incidental remarks which reflect credit on the author's judgment and learning.

A work of the above description is much wanted, on account of the scantiness of our present information on the Walcheren fever, and the discordant statements hitherto given, leaving the philosophic mind but little reason to be satisfied, and affording it but little ground for reflection. We are glad that the ideas which the author imparts to his readers, do not appear to have carried him beyond the limits of his personal experience, which seems every where corrected by his own good sense and acute observation. Dr. D. is already known by his French work, on the diagnosis of death and precipitate burial, and in the literary world by his History of Nice, &c. &c.

The author begins this work with an ample introduction on the Medical Topography of Zealand, and considers philosophically all the local and general causes which conspire to render the island of Walcheren so insalubrious. "Much of the tendency to disease in Zealand, and in Walcheren, seems to depend on local causes on its sea-line; for it has been confidently asserted by several writers, that there is a peculiar species of damp exhalation, which rises from the slime and mud laying on the beach, during the recess of the tide, and which has been thought more liable to produce bad effects, (for moisture always debilitates the body, and disposes it to disease,) from some chemical causes supervening from the mixture of the drainings of the land with the salts deposited on it, and, perhaps, with a portion of that putrescent phosphoric matter, which is known at times to be copiously distributed over the surface of the ocean."

Many curious circumstances are noticed, whereby the unhealthiness of the season may be detected, by observing the degrees of moisture beneath and upon the surface of the soil. He shews, (what few persons are inclined to deny) that bad water, exposure to inclemencies of the weather, fatigue, and moisture, all concurred to predispose the troops to fever, but that marsh miasmata were necessary to call the disease into action. The unhealthiness of the island of Walcheren, he evidences in the constitutional character racter of the inhabitants. "The general constitution of the adult here, is that to which ancient physicians have given the name of phlegmatic. The organization, in short, is feeble, the complexion sallow, the body bloated, and frequently anasarcous, and all the grand functions of life are weakly and incompletely performed. The women, like the men, have a natural weakness of constitution, and become old at an early period of life."

Here follows from the public documents, detailed reports of the sick, their state at Colchester and Ipswich, and a narrative of the author's appointment. He has seen the same fever in some French troops at Argues Martes, and in Piedmont.

We learn at the conclusion of the introduction, and in the two first sections, that the common type of the Walcheren intermittent, as it appeared in England, was the double tertian, and meet with some interesting inquiries into that very important point, the appearance of the fever, posterior to the arrival of the sick on our own shores. The author conceives this to be rare. He does not, however, deny that it may declare itself in individuals returned to England, who were well while abroad; and admits that there is no fixed period for marsh miasmata to operate on the system, of which the instances wherein the fever has not appeared till after the return of the troops to England, are proofs. "There is not that I know of, any particular and definite period for the reception of marsh exhalations into the system, and their action upon it. They have occasionally been found not to operate until weeks after the person has been exposed to their influence. There are certain symptoms, which, were they accurately noticed, such as debility, an oppressio virium, nausea, and head-ache, with which men were frequently seized before the fever appeared, indicative, in my opinion, of the difficult admission of the miasmata into the system, which seemed to await for some favorable change; as that, for instance, occasioned by exposure or irregularity of any kind, that would favour their entrance into, and operation on it. In every instance of disease, as far as I could ascertain, that fell to my lot to inspect, remission or intermission of the fever more or less evident, occurred, and I was left ignorant of this, its true appearance and character, from the imperfect representation of the sick; it was usually confirmed in the presence of those consequences which it is known invariably to entail. Admitting, however, that the fever had not, in every case, manifested itself in Walcheren, it merely proves, that with a predisposition existing after the application of the exciting cause, the constitution, under particular circumstances, had the power of suspending the action of miasmata for a time, but ultimately yielded to their influence when such a power ceased to act."

The author proceeds to give a definition and explanation of the type of the fever; first observing, that it assumed the quotidian, tertian, double tertian, quartan, and even remitting type; of all these, the double tertian was the most common, frequently forming the *anticipating and retarding intermittent*, by the hastening or protraction traction of the return of the exacerbations. "Sometimes a period of the fever," he observes, "was regular, that is, during the period there was but one paroxysm and one interval." Generally, however, he says, "instead of one paroxysm and one interval in each period, there were two intervals and two paroxysms, differing, however, materially, as to the violence and duration of symptoms, the hours of invasion, time of decline, and termination." He mentions, "that this type variously anticipating and p stponing, gave a peculiar mixed character to the disease, rendering it anomalous in nature, and tedious to the practitioner to treat." He remarks too, "that there was a variety of the disease, in which, on one day, there were two paroxysms, and on another, only one, while in other species of the fever, there has appeared to be a constant succession of paroxysms, one beginning as soon as a preceding one had ended." The author shews that the fever anticipated so much in some cases, that so many hours were gained, as to give a new character to the type, and even to make it resemble remitting more than intermitting fever. He dwells with great exactness on its irregular tendencies, and shews discernment in representing all the varieties of the double tertian type. The author confirms the observation of Clark, Fordyce, and Cleghorn, of the retarding paroxysm frequently failing to return in the night time, and of occupying again, in succession, the diurnal hours as before; a circumstance that he thinks contributed to change and modify the type anew.

In the next place, the author enumerates the irregularities which occasioned the double tertian type to change its course, and to become confused. Amongst these, we *find debility*, prolongation of the paroxysm, and visceral disease mentioned. He considers that these were the principal causes of such accidents, and very rarely an inflammatory diathesis. When, however, this did prevail, its force seemed much directed against the viscera of the abdomen, constituting a disease which the ancients called *assodes*.

The following section is devoted to an investigation of particular phenomena. The author's observations on the pulse are ingenious, and contain an account of various distinctions in the pulsations of the artery, which are applicable to the subject of fever in general.

The inferences he draws from the state of the brain, the tongue, the skin, the bowels, from external signs, and various phenomena, the secretions, &c. are all deserving of the highest consideration, and aid materially in forming a true prognosis. The whole of these are interspersed with appropriate illustrations. In the pathological view of the fever, the author takes some pains to shew that through the medium of nervous agency, marsh miasmata may produce vascular derangement, and this, at length, terminate in plethora of the venous system, which the author is inclined to believe is the cause of the torpor that precedes the exacerbation. "That venous plethora seems to have its origin in diminished nervous energy, is rendered probable by the dejection of spirits, the loss of muscular lar power, and the general debility which attend this fever in all its stages; also by the tendency there is in certain parts to be attacked with spasm when this disease inclines to a fatal termination. Such appears to me to be the cause of congestion in those organs whose vascular system is almost wholly venous, and in which the circulation is naturally slow, and liable to interruption." The author admits that torpor may be also occasioned by debility; but as he always found the venous system turgid with blood, at whatever period of the fever the patient died, he conceives this plenitude may, in its turn, become a cause of torpor, and ultimately of a paroxysm. The ratio symptomatum is methodical and excellent.

The predisposing, concurring, and exciting causes, occupy another section; after which the treatment of the fever is noticed. This, the author comprises principally under the head of tonic, purgative, and mercurial. We refer the reader to the numerous remarks he has made on cold affusion, tobacco, opiates, ligatures, emetics, bleeding, and various other expedients, to shorten the fit, and render it less violent. The numerous complications of the fever, and its combination with visceral disease, rendered bark a very ineffectual, nay, sometimes an injurious remedy. The author found there was no security against a relapse, nor any hope indeed of recovery, but where mercury was had recourse to, and persisted in for a great length of time, until all induration and vestige of visceral disease were removed. He also considers the efficacy of drastic purges so great, as even to rival the power of mercury in many instances. Upon this subject, he observes, that "far from thinking that drastic purgatives proved ultimately injurious, by favouring relapses, as Fordyce and Gregory the elder, pretend: I have no hesitation in saying, that when they were repeated at proper intervals, they not only diminished the tendency to relapse, but carried off the congestion of the abdominal viscera when other remedies failed; and proved the most effectual preventive of dysentery, arising from chronic inflammation of the intestines, a state that first induced diarrhœa, and always ended in that disease." He says too, "I am inclined to think that drastic purgatives, in a great number of instances, as the cases I shall insert will shew, were often more serviceable than mercury, in diseases of the spleen, liver, and intestines." Again, "I invariably found that those cases which were treated with drastic purgatives, were rendered milder and more tractable, and ceased infinitely sooner than the others: nay, I observed that while some patients were sinking under the use of bark and mercury together, others taking purgatives and mercurials were fast regaining health." The author condemns the indiscriminate and free use of mercury, and thinks that much harm has arisen in the practice of medicine, from doing what is quaintly called *pushing the mercury*. He condemns bleeding, except in particular cases. Observations then follow, on the employment of the auxiliary remedies, iron, nitric acid, and sulphate of copper. It is not in our power to make extracts of his remarks on all the various remedies he employed. We must, therefore, again request the reader to supply this deficiency, by a reference to the work itself.

From the treatment, the author proceeds to speak of the dreadful consequences entailed upon the constitution by the primary fever, which, independent of organic disease of the liver, spleen, and other viscera, we find to consist in diarrhœa, dysentery, ascites, anasarca, hydrothorax, hydrops-pericardii, anasarca pulmonum, dropsy of the brain, and jaundice. He takes occasion here to consider in a very ample manner chronic dysentery, which he refers to his favourite theory of venous plethora of the abdominal viscera, and sometimes to a vitiated quality of the bile, which to us appears to be the best founded opinion. He supposes that dysentery may originate from the same cause as fever, (which few are inclined to contest,) and dwells upon the success that attended the use of drastic purgatives in averting this worst and most irremediable termination of the disease. He mentions that the diarrhœa which preceded chronic dysentery, was only to be removed by drastic purgatives. " I was latterly so satisfied of the efficacy of drastic purgatives in these chronic diarrhœas, accompanied with obstructions of the liver and spleen, that I, at their first developement, instantly proceeded upon this treatment; and do not entertain a doubt, but that I frequently thus prevented dysentery from supervening." Patients either died dysenteric, dropsical, or expired from debility. It was no uncommon circumstance for a protracted and violent paroxysm to be followed by a sudden effusion in the chest, in which case the fever had rather a continued form than the intermitting type. " But the phenomena indicatory of effusion in the thorax, have not always come on gradually, and been combined together; for they sometimes, after a severe paroxysm of ague, invaded suddenly and with severity, putting an end to the patient's life in thirty-six hours. Nay, I have known men who were convalescents, attacked unexpectedly with a paroxysm of ague, which has terminated in effusion into the chest, and quickly destroyed the patient." The author next informs us, that one of the most formidable irregularities of this disease was pneumonia. In the preceding sections, he dwells at length on other irregularities, as congestion of blood in the liver and spleen, inflammatory affections of the abdominal viscera and diarrhœa; but he considers pneumonia as a combination with the primary fever, rather than a consequence of it, and proposes venesection as the only remedy, even in advanced stages of the Walcheren fever.

The book concludes with particular dissections, and a general view of the morbid anatomy of the body, a part which is extremely valuable, and arranged with precision and perspicuity. For the convenience of the reader, the whole of the morbid appearances are brought together, and the ravages committed by the fever on the viscera of the head, chest, and abdomen thus presented at one view. This is far too copious for us to make extracts from. We shall, however, observe, that it would have contributed much to

 the the completion of this useful book, had a few plates been introduced, illustrative of the morbid anatomy.

It is impossible, in the narrow limits assigned to a review, to enter more fully into the merits of this work. Suffice it here to say, that the fever and its consequences are traced with care and accuracy, that we consider it a valuable acquisition to a medical library, and recommend the perusal of it to our readers. It seems calculated, like the works of Cleghorn and Sir John Pringle, to be particularly worthy the attention of military practitioners. The whole book is interspersed with illustrations, and contains more research than could have been expected in the short time the author has had to compose it.

---

*A new Family Herbal, or Popular Account of the Natures and Properties of the various Plants used in Medicine, Diet, and the Arts.* By Robert Thornton, M. D. 8vo. pp. 888. London.

Many doubts have arisen on the utility of popular works on medicine, and the subject has, at various times, undergone much discussion; yet the question may still be considered as in a great measure undecided. For our own part, we confess, we do not think a science which, of all others, requires the extensive exercise of the highest powers of the mind to attain, and the practical application of which is admitted to be so difficult, can by any possibility be communicated in a compendium. When men who devote their whole lives to the cultivation of medical science, and who possess a fair share of abilities, and who are not deficient in industry and application, so often fail in their attempts to discover the real nature of a disease, and are frequently foiled in their endeavours to remove it, how shall we expect so difficult a task to be accomplished by a superficial acquaintance with a few general rules which are founded in empiricism, and calculated rather to mislead, than to inform those who consult them? While, however, there is such a propensity in mankind to quackery, and the practice of dabbling in medicine is so prevalent, we should be glad to see it productive of the least possible mischief; and surely a step would be gained, if the deleterious metallic poisons, such as calomel and arsenic, were banished from domestic use, and the good Lady Bountifuls were again to be employed in culling simples for possets and sovereign ointments. On this account we congratulate Dr. Thornton on the appearance of his book, which is calculated to inspire in its readers, a taste for botanical pursuits, and to attract their attention to the medical virtues of the vegetable tribes, which have perhaps, especially our own indigenous plants, been too much overlooked and neglected, even by physicians themselves.

The work is dedicated to Dr. Duncan of Edinburgh, and we shall transcribe the following passage in the dedication, as in some degree explanatory of the nature of it, and of the author's plan in the arrangement of his subject.

"A desire to become acquainted with the virtues of plants seems to have been coeval with the first dawn of knowledge; but the figures contained in the books treating of these subjects are so inaccurate, and the descriptions so vague, credulous, and, in every sense, so gross and vulgar, that mistakes are unavoidable, and false properties were bestowed on the most common and trivial plants.

"The Medical Botany of the ingenious and able Woodville cleared much rubbish from this Augæan stable, but the expensive mode of its publication deterred many practitioners, and families in general, from the purchase; there was, therefore, wanted for general and ordinary use a companion to your useful and perfect *Pharmacopœia.* Nothing more was required than simply to tread in your foot steps, adding figures by such an artist as Bewick, and correct descriptions, with the addition of some general prescriptions, combining at the same time from all authors whatever related to the subject. This could not be accomplished in a *pharmacopœia*; the present work, therefore, is presented to the world as a more complete and perfect herbal than has hitherto appeared; and as intended to unite the various advantages that have been derived to science from your 'Edinburgh New Dispensatory.' I take this opportunity, therefore, to acknowledge the source of much of my information, which I would not, indeed, disfigure by a change of words, but have generally transcribed from your work, so that considerable part of the merit which may be found in this Herbal must in justice be ascribed to your industry and intelligence; and I hope and trust, that the very superior engravings of Bewick will render it in every respect a useful introduction to Pharmaceutical Science."

Although the Doctor was unwilling to disfigure that portion of information he has transcribed from the Edinburgh Dispensatory, by a change of words, we do not think that enclosing it by inverted commas would have had that effect; and his readers would thereby have been better enabled to distinguish what is his own from what he has derived from that source. In many cases, perhaps in all, as the herbal is intended for a companion to the dispensatory, a reference to the latter book would have been preferable to a long quotation, which when frequently repeated, contributes to swell the volume to rather an enormous size. The same remark applies to the prescriptions, and with redoubled force, since the formulæ are copied from the London Pharmacopœia of 1787, although the new one had appeared some months previous to the publication of Dr. Thornton's book.

The doctor has also added the culinary properties of many vegetables, and has enriched his book with numerous recipes for making wines and other domestic preparations from the various plants he has described, all which cannot fail of being acceptable to the class of readers for whom his work is principally intended: at the same time, it would be injustice to deny, that much valuable information information on the medical virtues of different substances may be obtained from it by the medical practitioner, who will often see the result of extensive experience, and numerous fair trials, accurately and candidly stated under the respective articles.

We give the following extract as a fair specimen of the plan and execution of the work.

"COMMON MEADOW SAFFRON.

"COLCHICUM AUTUMNALE.

"*Class* VI. Hexandria. *Order* III. Tryginia.

"ESSENT. GEN. CHAR. *Corolla* six-parted: *Tube* radical: *Capsules* connected, inflated.

"SPEC CHAR. *Leaves* flat, lanceolate, erect.

"DESCRIPTION.—The root is a double succulent bulb. The flower is large, of a purple colour, and comes directly from the root. The leaves appear in spring, and are radical, and spear-shaped. Corolla consisting of a single petal, divided into six lance-shaped erect segments. Capsule three-lobed, divided into three cells, containing globular seeds, which are not ripened until the succeeding spring, when the capsule rises above the ground upon a strong peduncle.

"HISTORY.—Meadow saffron is a perennial bulbous-rooted plant, which grows in wet meadows in the temperate countries of Europe. It flowers in the beginning of autumn, at which time the old bulb begins to decay, and a new bulb to be formed. In the following May the new bulb is perfected, and the old one wasted and corrugated. They are dug for medical use in the beginning of summer. The sensible qualities of the fresh root are very various, according to the place of growth, and season of the year. In autumn it is inert; in the beginning of summer, highly acrid: some have found it to be a corrosive poison; others say they have eaten it in considerable quantity without experiencing any effect. When it is possessed of acrimony, this is of the same nature with that of garlic, and is entirely destroyed by drying.

"MEDICAL VIRTUES.—Stoerck, Collin, and Plenk, have celebrated its virtues as a diuretic in hydrothorax and other dropsies. But it is at best a very uncertain remedy. The expressed juice is used in Alsace to destroy vermin in the hair.

"From various observations on the effects of colchicum made by baron Stoerck, and especially upon the infusion of three grains of the fresh root in four ounces of wine, he remarked that its diuretic power was very considerable, and therefore concluded, that if its deleterious acrimony were destroyed, it might prove in this character an efficacious medicine: accordingly, he digested an ounce of the recent root, sliced in a pound of vinegar for forty-eight hours with a gentle heat; the vinegar being then strained, it proved acrid to the taste, constringed and irritated the fauces, and excited a slight cough; to obviate which, he mixed the vinegar with twice its weight of honey, and gently boiled it down to the consistence of honey, forming an oxymel sufficiently grateful; and which, which, taken in doses of a drachm, promoted a copious discharge of urine, without producing any inconvenience from its acrimony, though it moderately stimulated the fauces, and absterged the mucus. Thus, like the squill, it was found both expectorant and diuretic; and the successful use of this medicine, in various hydropic disorders in the hospital at Vienna, equalled the baron's utmost expectations. He recommends, at first, a drachm of the oxymel to be given twice a day in any suitable vehicle, and gradually to increase the dose to an ounce or more in a day. Many other practitioners, who employed the oxymel colchici in these complaints, also experienced its good effects, especially in Germany and France, where it continues to be a favourite medicine: in England, however, the colchicum has been less successful, and is very generally thought a less efficacious diuretic than the squill, which excels it still more as an expectorant. The London College, conformably to the practice of Stoerck, directs an oxymel colchici, and that of Edinburgh a syrup; the latter, however, differs from the former only in using sugar instead of honey.

"Preparation.—Syrup of Colchicum. (Syrupus Colchici Autumnalis. E.)

"Take of colchicum root, fresh, cut into thin slices, one ounce; vinegar, sixteen ounces; double refined sugar, twenty-six ounces: Macerate the root in the vinegar two days, occasionally shaking the vessel; then strain the infusion with gentle expression. To the strained infusion add the sugar, and boil a little, so as to form a syrup.

This syrup seems to be the best preparation of the colchicum. We must take care to gather this root in the proper season; and from [to] errors in this particular we are to ascribe the uncertainty in the effects of this medicine as found in the shops. It is chiefly employed as a diuretic, and may be taken from a drachm or two to the extent of an ounce, or more.

"Oxymel of Meadow Saffron. (Oxymel Colchici. L. D.)

"Take of the fresh root of meadow saffron, cut into thin slices, one ounce; distilled vinegar, one pint; clarified honey, two pounds by weight. Macerate the root of meadow saffron with the vinegar in a glass vessel, with a gentle heat, for forty-eight hours. Strain the liquor, pressed out strongly from the root, and add the honey. Lastly, boil the mixture, frequently stirring it with a wooden spoon, to the thickness of a syrup. This is an active preparation, but its use may be entirely superseded by the syrup of the same root. The dose given is a drachm to half an ounce."

Every plant described is accompanied by an engraving; these, as may be expected, in so numerous a list, are of unequal value, some very accurately representing the plant for which it is designed, and others being not quite so fortunate. The articles follow according to their botanical arrangement, and the want of an index is much felt, for he who wishes to see the description of any paticular plant, and is unacquainted with its class and order, has no other other resource but the running title to guide him in his search; this is certainly no small inconvenience in consulting a bulky volume like the present one. It is not our intention to search for and detail little errors and inaccuracies, for such must occur in every book of considerable size, but we certainly were a little surprised by the Doctor's remark, (p. 20.) that Turner's Cerate received its appellation because it is used in curing *the wounds of turners;* we had always thought it derived its name from the Inventor, who first recommended it as possessing very healing qualities, on which account it was adopted by the London College, under the title of Ceratum Epouloticum. We do not however often meet with similar errors, and are persuaded this must have escaped the author's notice.

We shall here finish our few remarks upon Dr. Thornton's work, and we hope they will be received in the same spirit in which they were written, as hints for the improvement of a future edition, which we have no doubt it will speedily attain; and we flatter ourselves that the author will coincide with us in opinion, that the book would not be injured, but rather improved by a careful and judicious curtailment, as well as a proper Index.

---

### Edinburgh Journal. No. XXII.

Article 1.—*Observations on the Digestion of the Stomach after Death.* By Allan Burns, Member of the Royal College of Surgeons in London, and Lecturer on Anatomy and Surgery in Glasgow.

Mr. Burns observes, that in dead bodies, the stomach has repeatedly been found eroded, where the previous symptoms gave no reason to suspect such an occurrence. This has been attributed by Mr. Hunter, to the action of the gastric juice, and Mr. Burns agrees with him in this opinion. Mr. Hunter, however, thought that the gastric juice, which was already poured into the stomach, effected this change. Mr. Burns seems to think differently, and states several cases wherein erosion had taken place at parts of the stomach, which could not have been acted upon after death by the contents of the stomach, and therefore that some other cause must be sought for.

He thus sums up his opinion: "If, then, the stomach was not acted on by fluid contained in its cavity, how came it to be dissolved? To me it appears, that we cannot, with propriety, ascribe the digestion of the stomach, in every case, to the gastric juice which has been poured into the cavity of that viscus. We are more properly to refer it, in some instances, to the action of the gastric fluid retained in the vessels which had secreted it. If this be admitted to be a correct explanation of the fact, that the fore-part of the stomach is sometimes digested by the gastric juice, we shall cease to have any difficulty in accounting for the dissolution of other parts of this viscus, besides the large end. We shall learn, that the part acted on must vary, according to the place of the stomach where where the gastric juice is retained in the apparatus which secreted it, and thus we shall be enabled to explain some cases, which, at present, seem to be in opposition to the observations of Mr. Hunter."

The author states some points, in difference between Dr. Adams and himself, upon this subject, who, he imagines, has mis-apprehended Mr. Hunter's opinion as to the state of previous health or disease, in which this erosion of the stomach could take place. It is superfluous for us to enter into this question, we shall refer our readers to a paper contained in a former part of this number, wherein Dr. Adams has entered pretty fully into the merits of it, and defended the statement he had given of Mr. Hunter's opinions. We shall only observe in conclusion, that we do not exactly coincide with the author in opinion, that "poison has been called in to account for the appearance; and thus the life of the person supposed to have administered the deleterious substance might, it is evident, be brought into danger."

We think there is not any medical practitioner, who would in a judicial investigation, attribute erosion of the coats of the stomach to poison, unless it was accompanied by evident marks of previous inflammation, or with gangrenous appearances; perhaps, in such serious circumstances, he would not positively affirm it without discovering some of the deleterious substance among the contents of that viscus.

Article 2.—*Regulations with regard to the passing of Conscripts for the French Army.* Extracted from the Code de la Conscription.

In France, there is not that discretionary power vested in the military surgeons, which exists in this country, of determining on the ability or disability for duty of individuals liable to military service; the regulations on this head, enacted by the government, are minute, and the execution of them are entrusted to the municipal and central administrations, who are to decide on the validity of the surgeon's opinions, and even the examinations of the conscript, are to be made in the presence of the administration, or if the conscript is unable to attend the Board, in that of a delegate from it. "Officers of health, and others convicted of having given a false certificate of infirmities or inabilities, or of having received presents or gratifications, shall be punished by not less than one, or more than two years imprisonment, or by fine, not less than 300, or more than 1000 francs."

The regulations by which the officers are to be governed in their opinions, are contained in two tables; the first, contains a list of "evident infirmities, implying absolute incapability;" the second is entitled "infirmities or diseases which occasion absolute or relative incapability for military service, and which are reserved for the examination and opinion of the central administrations of the department." Some of these infirmities, only entitle to a provisional exemption, and every care seems taken to guard against abuses of indulgence.

The

The following passage will convey an idea of the rigid rules which are observed in the examination of conscripts.

"We know that young people in the country are more subject to those affections, (rheumatic) than those in towns, and that in some kind of abodes, they are more easily contracted. Joining all these data, combining and comparing them together, the surgeons may commonly distinguish a real affection from a feigned one. As it is but just that, in some other equivocal cases, such as those respecting the diseases of the breast, humanity should incline to the conscript's side; so, with respect to pains and rheumatisms, which are not proven, it is equally proper to prefer severity to indulgence, as military exercise, far from aggravating this predisposition, if it exists, will only contribute to remove it."

ARTICLE 3.—*Letter from Professor E. M. of Rosenschoeld, of the University of Lund, in Sweden*, to C. CHISHOLM, M. D. F. R. S. &c. Communicated by Dr. Chisholm.

IN a former Journal, p. 166, we gave an account of a paper on the Lues Bovilla, by Dr. Chisholm, to which this may be considered as a supplement. It consists of certain questions, addressed by him to Dr. Florman. Professor of Anatomy in the University of Lund, in Sweden, together with the Professor's Answers. Dr. Florman himself has never seen this disease, it not having occurred in Sweden since the year 1766, nor in Denmark since 1772; "but to judge from the best Swedish and Danish descriptions of the disease, it comes nearest to the Pestis Bovilla of Lancisci, and to the Lues Bovina described by Ramanzini and Camper." The pustular eruption, however, has not been a constant symptom in those northern climates, but more frequently wanting. Although the pestis bovilla has not appeared in Sweden for so long a period, a disease somewhat similar frequently occurs, which the author describes as a malign fever, united with anthrax, which most often kills before the third day. He says, "this disease is very different from the genuine pestis bovilla, and I have never seen or heard of the latter arising from the former. It prevails in the warmer summer months, and ceases of itself in the autumn or sooner." It seems to depend upon local circumstances, and arises in some places without having been brought to them by contagion. Having in our former remarks pretty fully considered Dr. C's opinions, we have little to add. We think, however, that the inference drawn by Dr. Chisholm, of the identity of the pestis bovilla in Europe, with the disease that prevailed in Grenada in 1783, requires further support to establish it completely. No circumstance is more worthy of remark, than that although such deleterious and fatal effects arise in the intertropical climates, from eating, or even handling the flesh of animals dying of the disease, no such effects take place in Europe in the true pestis bovilla, where the disease is entirely confined to a single species of animal, while yet the malign fever with anthrax in Sweden, frequently attacks at the same time horned cattle and horses, nay, also the human species: this was precisely the case when when the cynanche maligna raged at Grenada in 1783, for Dr. C. does not fail to remark its concomitancy with a contagious distemper epidemic among the cattle and mules in the same parts of the island where the cynanche maligna appeared.

We subjoin the following quotation on the subject, of the resemblance of the pestis bovilla with the small-pox.

" Q. *5th.* Are there any good reasons for believing, that the Pestis Bovilla partakes of the nature of variola or small-pox; and that the disease may be communicated like it by inoculation? And have any attempts been made in Sweden or Denmark to ascertain this?

" *A.* I cannot find that the true Pestis Bovilla corresponds with variola in mankind. At least, the elevated pustule ought to be, in that case, a more constant and general symptom of this plague. It can, nevertheless, as other contagious complaints, be propagated by inoculation, of which we have a number of examples, as well in Holland as in Denmark, where many of the inoculated cattle had recovered, and since not been contaminated by other sick cattle. For the inoculation has commonly been used the matter which discharges from the nose of the sick cattle."

ARTICLE 4.—*Case of disordered Alimentary Canal, supposed to have arisen from the Person swallowing a Skein of Cotton several Years before.* By Mr. FINCHAM.

A lady of 25, accidentally swallowed a skein of cotton; no inconvenience was felt for three months, when upon being electrified, she was seized with violent pain in the stomach, without sickness; the pain returned every day for some months. From a friend's only requiring her to take food, says the author, she became sick, and vomitted the cotton, which, from being white, had acquired a permanent green colour, but was little changed in texture; much general indisposition remained for seven or eight months, but she afterwards enjoyed good health.

Then follows a diary of the symptoms and mode of treatment of a case of constipation, in which we can trace no connexion with the skein of cotton that had been previously swallowed, but in which we clearly perceive the beneficial effects of the purgative plan of treatment carried to some extent, as recommended by Dr. Hamilton. " May 17. The bowels were relieved in the night of the 15th and yesterday morning, of hardened fæces to a prodigious amount." Blister after blister was applied to the stomach, and small doses of calomel (that modern panacea) with hyoscyamus, were given in pills; it was only, however, by repeated purgatives, that relief was afforded and amendment produced. " 23d, a senna mixture was substituted for the pills." " 25th, did not begin the mixture till noon yesterday. She has had three copious evacuations, the first extremely offensive. This operation has produced considerable amendment." It is unnecessary to detail the remainder of the case, as it consists of similar reports; amendment when the bowels were kept free, and, as might be expected, aggravation of the symptoms when this was at any time neglected.

ARTICLE

ARTICLE 5.—*On the Treatment of Ganglia by Escharotics.* By JAMES WOODHAM.

"GANGLIA, says the author, though not frequent, are far from being uncommon; and as they always prove inconvenient to the patient, and sometimes troublesome to the surgeon, under the means usually employed for their removal, the annexed cases, treated successfully by a mode somewhat novel, will not, I trust, be unacceptable to the profession."

Two cases are given, in which the treatment consists in applying a blister to the tumour, removing the detached cuticle, and plentifully sprinkling the cutis with the oxyd of arsenic in powder, and repeating this every day, till a complete eschar was formed. To procure a separation of the eschar, and to remove the inflammation that had been excited, an emollient poultice was applied morning and evening. In ten days the eschar was completely formed; in a fortnight it was thrown off, leaving a healthy ulcer the size of a shilling, which was daily dressed with an ointment of wax and oil, occasionally sprinkling it with nitric oxyd of quicksilver. The whole process of cure took up in each case, something less than three months.

ARTICLE 6.—*Observations upon Ulcers of the Leg.* By JOHN WEBB, Surgeon.

THE author has in this paper presented to us some observations, with an attempt to explain the principle which should be kept in view in the treatment of the irritable ulcer. The worst cases, he says, which have come under his care, have been attended with the following character: "the constitution is affected with debility; the digestive powers are impaired; the function of the liver is diseased; the circulation is hurried; an anxious watchfulness is depicted upon the countenance; and, from the general irritability which pervades the whole constitution, the patient is seldom relieved by the soothing tranquillity of sleep." His peculiar treatment we shall give in his own words.

"The motives which induced me to depart from the regular routine of practice, were from a case where every known application had been tried, without procuring the wished for effect. Here the constitution was greatly disordered. In this case, the applications which alleviated the sufferings of the patient most, were fomentations and poultices. Seeing the sufferings of the patient were quelled for a certain time, I was led to consider why the poultice should act thus beneficially for *a time*, and no longer, and this whether the application was warm or cold. The conclusions which I drew, were, that the *secreted pus* must act as an irritating cause, and the patient was easy as long as the poultice *absorbed the secreted pus*, and kept the surface of the sore clean, and not from the sedative effect of the application, as is generally supposed. The next visit, I dressed the ulcer with two folds of dry lint, over which linen was to be kept wet, with an evaporating lotion, to allay the surrounding inflammation, inflammation, and keep the edges of the lint from adhering to the sides of the ulcer, which was to be removed immediately when the patient experienced any pain, and fresh dressings to be substituted. The success which attended this practice, surpassed my most sanguine expectations. In the short space of ten days, an immense chasm was filled with strong healthy granulations; the edges under this treatment were kindly cicatrizing; a diseased constitution was rapidly restored."

( To be Continued. )

